# Editorial: Unveiling the potential of beneficial microbes and their metabolites: a comprehensive approach to plant health

**DOI:** 10.3389/fmicb.2026.1994278

**Published:** 2026-09-25

**Authors:** Yaqian Li, Yi Zhou, Yunpeng Chen, Bolin Zhang

**Affiliations:** 1 School of Agriculture and Biology, Shanghai Jiao Tong University, Shanghai, China; 2 State Key Laboratory of Microbial Metabolism, Shanghai Jiao Tong University, Shanghai, China; 3 School of Agriculture, Food and Wine, The University of Adelaide, Waite Campus, Urrbrae, SA, Australia; 4 College of Biological Sciences and Biotechnology, Beijing Forestry University, Beijing, China

**Keywords:** beneficial microorganisms, biological control, microbial metabolites, plant growth promotion, plant–microbe interactions, sustainable agriculture

Agriculture faces a dual imperative: sustaining crop productivity while reducing reliance on chemical fertilizers and pesticides under increasingly variable environmental conditions. Beneficial microorganisms are central to this transition because they can improve nutrient acquisition, suppress pathogens, enhance stress tolerance, and influence plant development. Their effects, however, cannot be predicted from taxonomic identity alone. Rather, they emerge from interactions among microbial traits and metabolites, host physiology, resident microbial communities, environmental conditions, and the formulation processes that determine whether an inoculant remains viable and functional after application. The 11 original studies assembled in this Research Topic address these interconnected dimensions, spanning microbial discovery and mechanistic characterization, plant–microbiome interactions and community ecology, product formulation, and field performance.

The first group of studies expands the biological resources available for crop protection while demonstrating why phenotypic screening should be complemented by mechanistic characterization. Han et al. investigated the predatory bacterium *Myxococcus fulvus* KS01 and showed that extracellular proteins, hydrolytic enzymes, and volatile compounds damaged *Verticillium dahliae*. A solid formulation subsequently reduced cotton Verticillium wilt and promoted seedling growth, linking antagonistic mechanisms to practical efficacy. Li et al. characterized *Pseudomonas shiyinii* sp. nov. ST4 and identified a PQQ-dependent glucose dehydrogenase system as the major driver of environmental acidification that disrupted mating in the sugarcane smut pathogen. Wang Z.-Q.et al. characterized *Paenibacillus* strain JDF1 as an antagonist of citrus bacterial canker and linked its activity and thermostability to a broad genomic and metabolic repertoire, including bacilysocin and other predicted antimicrobial compounds. Despite their distinct modes of action, these studies collectively demonstrate the value of integrating ecological discovery with genome-informed taxonomy, functional characterization, and validation in plant systems.

Several contributions focus more directly on microbial metabolites as mediators of plant protection and growth promotion. Sahu et al. integrated untargeted metabolomics, genome mining, and colonization imaging to associate the endophytic competence of *Stenotrophomonas maltophilia* 3A with diverse secondary metabolites and biosynthetic gene clusters relevant to tomato health. Wang E.-X. et al. provided causal evidence that siderophore production by the nitrogen-fixing bacterium *Kosakonia radicincitans* GXGL-4A contributes to both cucumber growth promotion and antifungal activity. Gan et al. developed a tiered strategy for selecting lipopeptide-producing *Bacillus* strains, quantified surfactin, iturin, and fengycin, and extended their evaluation from antimicrobial assays to crop responses under field conditions. Together, these studies move strain selection beyond inhibition-zone measurements alone. Candidate inoculants can be evaluated more rigorously when defined metabolites, effective colonization, and reproducible plant responses are connected within the same experimental framework.

The Research Topic also moves beyond the assumption that a single organism or active compound will be sufficient under field conditions. Bai et al. combined metabolites from *Trichoderma asperellum, Metarhizium anisopliae*, and *Bacillus subtilis* in a biochar-supported formulation. When integrated with reduced pesticide doses, the formulation controlled multiple rice diseases and insect pests under field conditions while reducing chemical inputs by approximately 30%. This study highlights an important principle for translation: biological products need not be positioned solely as direct replacements for conventional pesticides. Instead, they can form part of integrated management strategies in which microbial products, suitable carriers, and compatible chemical inputs are combined to improve stability, consistency, and operational feasibility.

A complementary group of studies examines the reciprocal relationship between plant metabolism and microbiome assembly. Ma et al. linked altitude-associated soil properties and microbial community structure with secondary-metabolite accumulation in *Lagotis brevituba*, revealing an environment–microbiome–metabolite relationship relevant to medicinal plant quality. Shan et al. followed *Rhododendron hybridum* from flowering to senescence and observed coordinated changes in root triterpenoids and rhizosphere microbial communities, accompanied by reduced network complexity and increasingly deterministic community assembly. Xun et al. associated contrasting grape responses to downy mildew with phenylpropanoid-related metabolites and transcriptional programs, emphasizing that host metabolic state is itself an important determinant of disease outcome. These findings broaden the conceptual scope of the Research Topic: beneficial metabolites are not restricted to compounds produced by inoculated microorganisms. Plant-derived molecules can also recruit, exclude, or reorganize microbial partners, thereby shaping plant health and disease responses.

Efficacy ultimately depends not only on biological activity but also on whether beneficial strains can withstand production, storage, and environmental stresses. Wang J. et al. showed that exogenous K^+^ enhanced desiccation tolerance and adhesion in *Pseudomonas protegens* SN15-2. Transcriptomic analysis and gene overexpression further implicated GDP-mannose 4,6-dehydratase in this response. This work addresses a persistent gap between laboratory antagonism and product performance: a microorganism with strong antimicrobial or growth-promoting activity may still fail in practice if it cannot survive processing, storage, rehydration, environmental stress, or competition following application.

Taken together, the contributions to this Research Topic outline a coherent development pathway for microbial crop protection and plant growth promotion: discover ecologically relevant microorganisms, identify the mechanisms and metabolites responsible for their activity, determine how these functions operate within the plant–microbiome–environment system, and develop formulations that preserve efficacy under practical conditions. This progression is a distinctive strength of the Research Topic. It treats beneficial microorganisms neither as isolated strains nor simply as sources of promising metabolites, but as living components of dynamic, context-dependent biological systems.

Looking ahead, progress will require comparable multi-stage screening pipelines, causal validation of microbial metabolites and host targets, and explicit assessment of dose, safety, persistence, and non-target effects. Multi-location and multi-season trials will be essential for distinguishing robust microbial functions from responses specific to particular soils, climates, crop genotypes, or resident microbiomes. Likewise, synthetic communities and metabolite combinations should be designed around ecological and functional compatibility rather than assembled solely from individually effective components. Longitudinal multi-omics and spatially resolved approaches will further help determine when beneficial interactions emerge, where they operate, and how long they persist. By more closely connecting mechanism, ecology, formulation, and field deployment, the studies in this Research Topic provide a foundation for developing more predictable and effective microbial interventions that support plant health and crop productivity while reducing dependence on chemical inputs.

